# Excitatory neurons in paraventricular hypothalamus contributed to the mechanism underlying acupuncture regulating the swallowing function

**DOI:** 10.1038/s41598-022-09470-9

**Published:** 2022-04-06

**Authors:** Si Yuan, Bing Deng, Qiuping Ye, Zhennan Wu, Junshang Wu, Lin Wang, Qin Xu, Lulu Yao, Nenggui Xu

**Affiliations:** 1 South China Research Center for Acupuncture and Moxibustion, Guangzhou Higher Education Mega Center, Medical College of Acu-Moxi and Rehabilitation, Guangzhou University of Chinese Medicine, 232 East Ring Road, Panyu District, Guangzhou, 510006 People’s Republic of China; 2 Department of Rehabilitation Medicine, The Third Affiliated Hospital, Sun Yat-Sen University, No. 600, Tianhe Road, Guangzhou, 510630 Guangdong China

**Keywords:** Neuroscience, Diseases

## Abstract

Paraventricular hypothalamus (PVH) is demonstrated to regulate stress, feeding behaviors, and other related homeostatic processes. However, no direct evidence has been investigated for the role of PVH in swallowing function. Acupuncture therapy at Lianquan (CV23) acupoint has been reported to improve the swallowing function in clinical trials, but its underlying mechanism still needs to be uncovered. Thus, we aimed to explore whether PVH involved the acupuncture mediated regulating swallowing function. Chemogenetics, electromyography (EMG) recording, and immunofluorescence staining methods were combined to demonstrate that neurons in PVH could be activated by electroacupuncture (EA) stimulation at CV23, and this neuronal cluster was represented as excitatory neurons. Furthermore, we mapped both the inputs and outputs of PVH neurons using viral tracing. The neurons in PVH projected with the brain regions, including parabrachial nucleus (PBN) and the solitary tract nucleus (NTS), which both participated in the swallowing process. The EA function regulating the swallowing was attenuated after inhibiting the neurons in PVH in the post stroke dysphagia. In conclusion, this study suggested that EA at CV23 could regulate swallowing function involving the excitatory neurons in PVH.

## Introduction

Swallowing is a critical process for allowing food and fluid to be ingested safely and efficiently, and thereby maintaining physiological and biochemical function. Dysphagia, which commonly occurs after stroke, is associated with the risk of death, occurrence of pneumonia, poor quality of life, and longer hospital stay^1^. Electroacupuncture (EA) therapy is an important and traditional intervention in dysphagia in China^2–4^. The treatment of dysphagia after stroke with acupuncture at Lianquan (CV23) has been documented in ancient Chinese medicine books, and its efficacy has been practiced in both pre-clinical experiments and the clinical trials^3, 5, 6^. However, the mechanism underlying EA at CV23 acupoint on swallowing function was still elusive.

The process of food intake depends on counts of chews and swallows^7^. It is known that solid food is transported to the pharynx actively in parallel to it being crushed by chewing and mixed with saliva in the oral cavity, which has to be mixed for a food bolus to reach the swallowing threshold^8^. Feeding behavior is regulated by neural circuits containing the hypothalamus and hindbrain in the mammals^9^. Paraventricular hypothalamus (PVH), which is located in the ventral diencephalon adjacent to the third ventricle, is essential to food intake^10^. Varela et al. suggested that the central lateral parabrachial nucleus (cLPBN) is highly innervated by fibers from the PVH, and the cLPBN-PVH circuit is necessary to induce feeding behaviors^11^. Recent studies indicated that arcuate nucleus (ARC)-PVH and ventral part of the lateral septal nucleus (LSv)-PVH circuits are also important in regulating the food intake behavior^12, 13^. Roman et al. reported that the stimulation of the nucleus of solitary tract nucleus (NTS)-PVH circuit signals induces appetitive behavior^14^. Thus, we hypothesized that neurons in PVH might also regulate the swallowing function.

Our previous studies have shown that EA treatment is effective for swallowing disorders. It suggested that EA at CV23 could increase the motor conduction velocity in hypoglossal nerve, enhance the electromyography (EMG) of mylohyoid muscle, and promote release of the substance P in the local acupoint^15^. Besides that, EA at CV23 could activate the swallowing related neurons in the primary motor cortex (M1), parabrachial nucleus (PBN), NTS, nucleus ambiguous (NA) and ventrolateral medulla (VLM)^2, 15^, and increase the expression of brain-derived neurotrophic factor^5^. However, no evidence showed the role of PVH in the acupuncture improving the swallowing function.

In the present study, we suggested that the excitatory neurons in PVH could be selectively activated by EA at CV23. The function of PVH is crucial for swallowing process and EA mediated efficacy in both physiological and pathological conditions. This study provided the fundamental and important insight of the EA at CV23 to treat swallowing related disorders for further clinical application.

## Results

### Effects of electroacupuncture at CV23 on c-Fos expression in PVH

**Figure 1:**
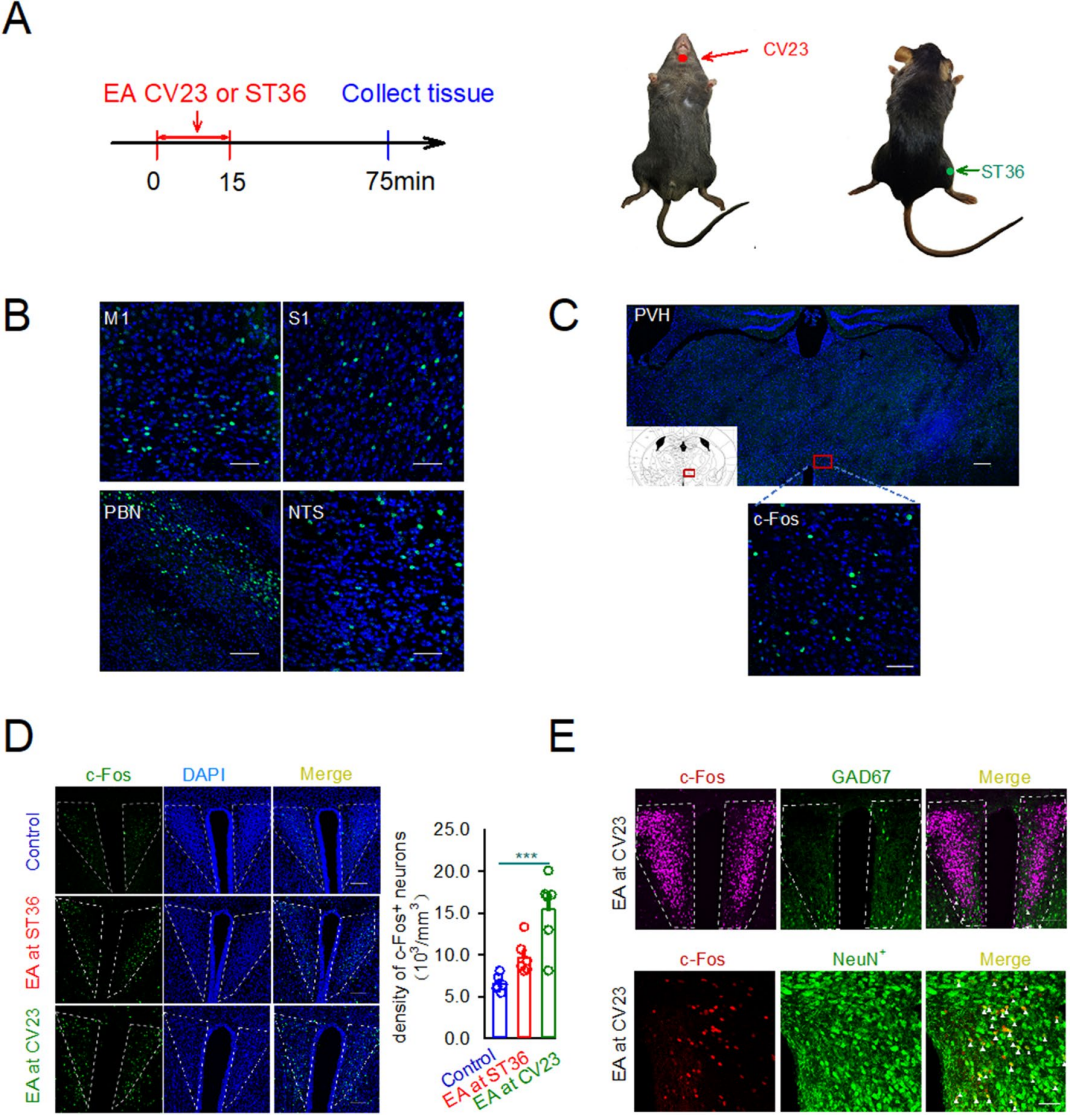
Effects of EA at CV23 and ST36 on c-Fos expression in PVH. (**A**) Schematics of experimental design and the CV23 acupoint and ST36 acupoint. (**B**) Confocal images showing c-Fos expression in M1, S1, PBN and NTS. PBN: Scale bars:100 μm. Others: Scale bars: 50 μm. (**C**) Representative confocal images of the c-Fos positive neurons in PVH activated by the EA Scale bars:1000 μm. Magnification is shown in the bottom, Scale bars: 50 μm. (**D**) Control group (top), ST36 group (middle), CV23 group (bottom). The statistical analysis of the density of c-Fos^+^ neurons in each group.10^3^/mm^3^. Data are shown as the mean ± SEM (N = 6 slices per group). Scale bars: 100 μm (**E**) Top: co-localization of c-Fos with the GAD 67, Scale bars:100 μm, bottom: co-localization of c-Fos with the NeuN, Scale bars: 50 μm. Arrows highlight the co-labeled cells. One-way ANOVA with Tukey's multiple comparisons test, ***p < 0.001.

c-Fos staining was widely used to label the neurons newly activated^16^. Electroacupuncture can induce c-Fos expression in brain regions (Fig. 1A). The results showed that c-Fos positive neurons in M1, the primary sensory cortex (S1), PBN and NTS increased by the EA at CV23 (Fig. 1B), which was consistent with previous studies suggesting these brain regions related to the swallowing process^17–19^. Besides that, we found that EA at CV23 induced the c-Fos expression in PVH (Fig. 1C). Furthermore, low c-Fos expression were observed in caudate putamen (striatum) (CPu), nucleus of the vertical limb of the diagonal band (VDB), medial lemniscus (ml) and pontine reticular nucleus, ventral part (Pnv), which has been reported to be few related with the swallowing function (Fig. S1A)^20, 21^. The density of c-Fos neurons in the PVH in the CV23 group were significantly increased, comparing to that in the Zusanli (ST36) group (Control: 6580 ± 387.8, EA at CV23 group: 15,441 ± 1730, cells/mm^3^, *p* < 0.001). There was no statistical difference between the control group and the ST36 group (Fig. 1D). These results suggested that PVH specifically responds to stimulation at CV23 acupoint. Compared with the responses to ST36 acupoint linking to the anti-inflammatory, pains and gastrointestinal disturbs^22, 23^, PVH was more sensitive to CV23 acupoint stimulation, which was thought to regulate the swallowing function (Fig. 1D). The anatomical studies have demonstrated the presence of glutamatergic neurons within the PVH, as well as occasional GABAergic neurons^24^. Double staining showed that c-Fos neurons in PVH activated by EA was co-labelled with NeuN^+^, a marker of neurons, while with few GABAergic neurons in the GAD67-GFP mice (Fig. 1E). Thus, our results suggested that the excitatory neurons in the PVH could respond to EA stimulation at CV23.

### Inputs and outputs of PVH neurons

**Figure 2:**
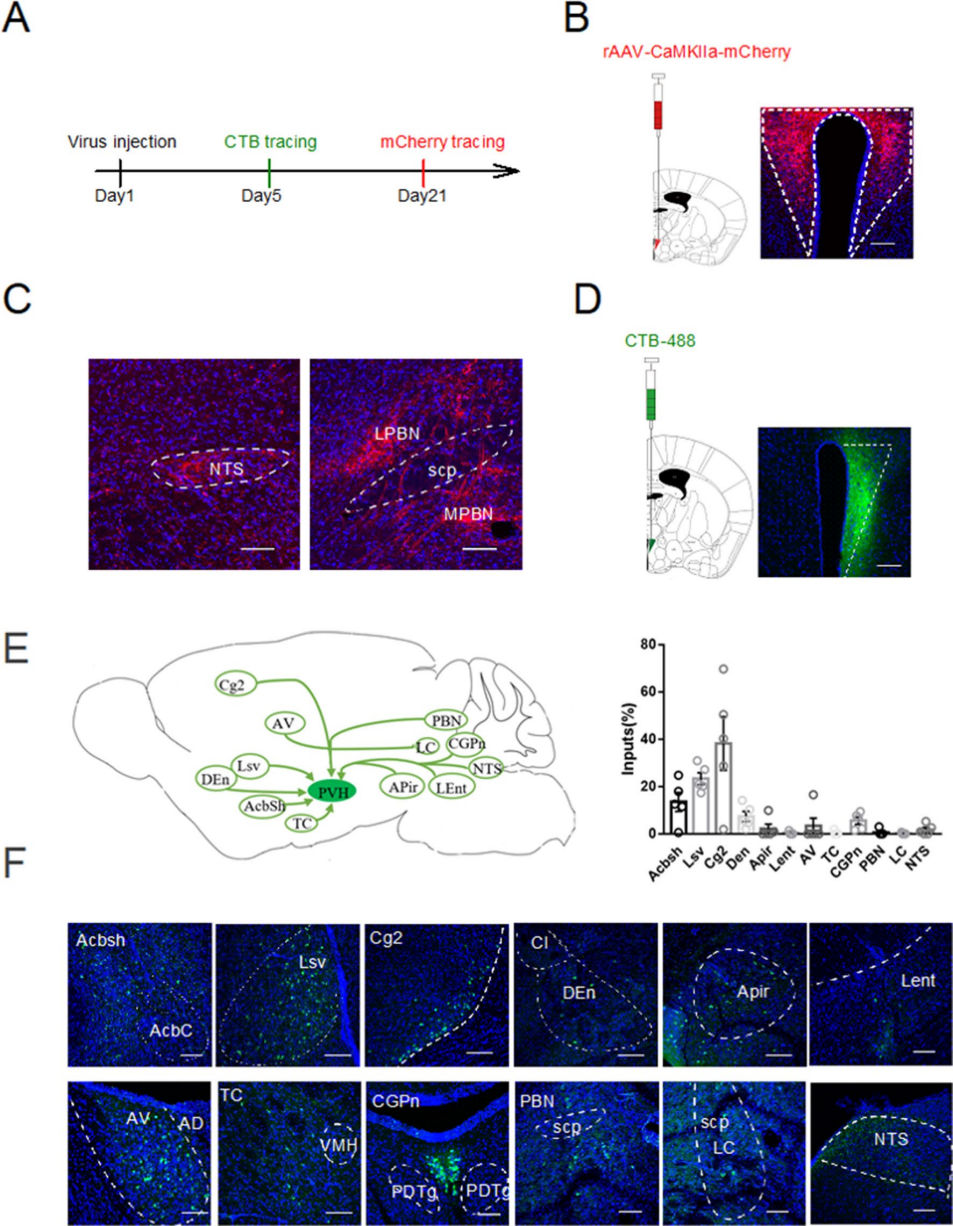
Inputs and outputs of PVH neurons. (**A**) Scheme for specific infection of projection neurons with CTB or mCherry. (**B**) Strategy to anterogradely label the brain regions receiving the projection from PVH by injecting the CaMKIIα-mCherry virus into the PVH (**C**) Confocal images showing mCherry^+^ fibers in NTS (left) and PBN (right) from PVH. Scale bars, 100 μm. (**D**) Left: Strategy to retrogradely label PVH afferents with CTB-488, Right: Confocal images showing CTB injections into PVH. Scale bars, 100 μm (**E**) Left: Cartoon images showing labelled neurons in select upstream regions in the whole brain, right: quantification of monosynaptic inputs in the whole brain, shown as the percent of labelled neurons from a given brain region relative to the total labelled neurons throughout the whole brain, (**F**) Confocal images showing labelled neurons in the selected upstream regions in the whole brain. AcbSh: accumbens nucleus, shell, AcbC: accumbens nucleus, core, aca: anterior commissure, anterior part, Lsv: ventral part of the lateral septal nucleus. Ventral part, Cg2: cingulate cortex, area2, CI: claustrum, DEn: dorsal endpiriform nucleus, APir: amygdalopiriform transition area, Lent: lateral entorhinal cortex, AV: anteroventral thalamic nucleus, AD: anterodorsal thalamic nucleus, TC: tuber cinereum area, VMH: ventromedial hypothalamic nucleus, PDTg: posterodorsal tegmental nucleus, CGPn: central gray of the pons, PBN: parabrachial nucleus, scp: superior cerebellar peduncle, LC: locus coeruleus, NTS: the solitary tract nucleus. Data are shown as mean ± SEM, N = 5 mice. Scale bars, 100 μm.

To further explore the other brain regions connecting to excitatory neurons in PVH, we mapped the monosynaptic outputs of PVH neurons using mCherry with CaMKIIα promoter (Fig. 2A,B). The mCherry^+^ fibers were observed in the NTS and PBN (Fig. 2C), which suggested neurons in NTS and PBN could receive input from the excitatory neurons in PVH. Notably, NTS contains the generator neurons involved in the triggering, shaping, and timing of the sequential or rhythmic swallowing patterns^25^, while PBN participates in mediating the dive reflex, laryngeal adductor control, swallowing function and upper airway tone, which acts as a mediator that relate primarily to laryngeal closure, upper airway tone and swallowing^26^.

We next aimed to explore how the neuronal population in PVH receives inputs from other brain regions. To solve this problem, we injected retrograde tracer CTB-488 into PVH to screen the monosynaptic projection inputs (Fig. 2A,D–F). The potential upstream targets of PVH neurons mainly consisted of cingulate cortex, area2 (Cg2), the accumbens nucleus, shell (AcbSh), LSv, dorsal endpiriform nucleus (Den), amygdalopiriform transition area (Apir), anteroventral thalamic nucleus (AV), central gray of the pons (CGPn), PBN and NTS. The activation in contralateral cingulate cortex was associated with a better motor recovery^27^. LSv is associated with food intake^13^. NTS and PBN is involved in the swallowing related process^17, 26^. All together, these results suggested the structural basis for PVH to play a role in function associated with the swallowing process.

### The function of PVH during EA treatment for poststroke dysphagia mice model

**Figure 3:**
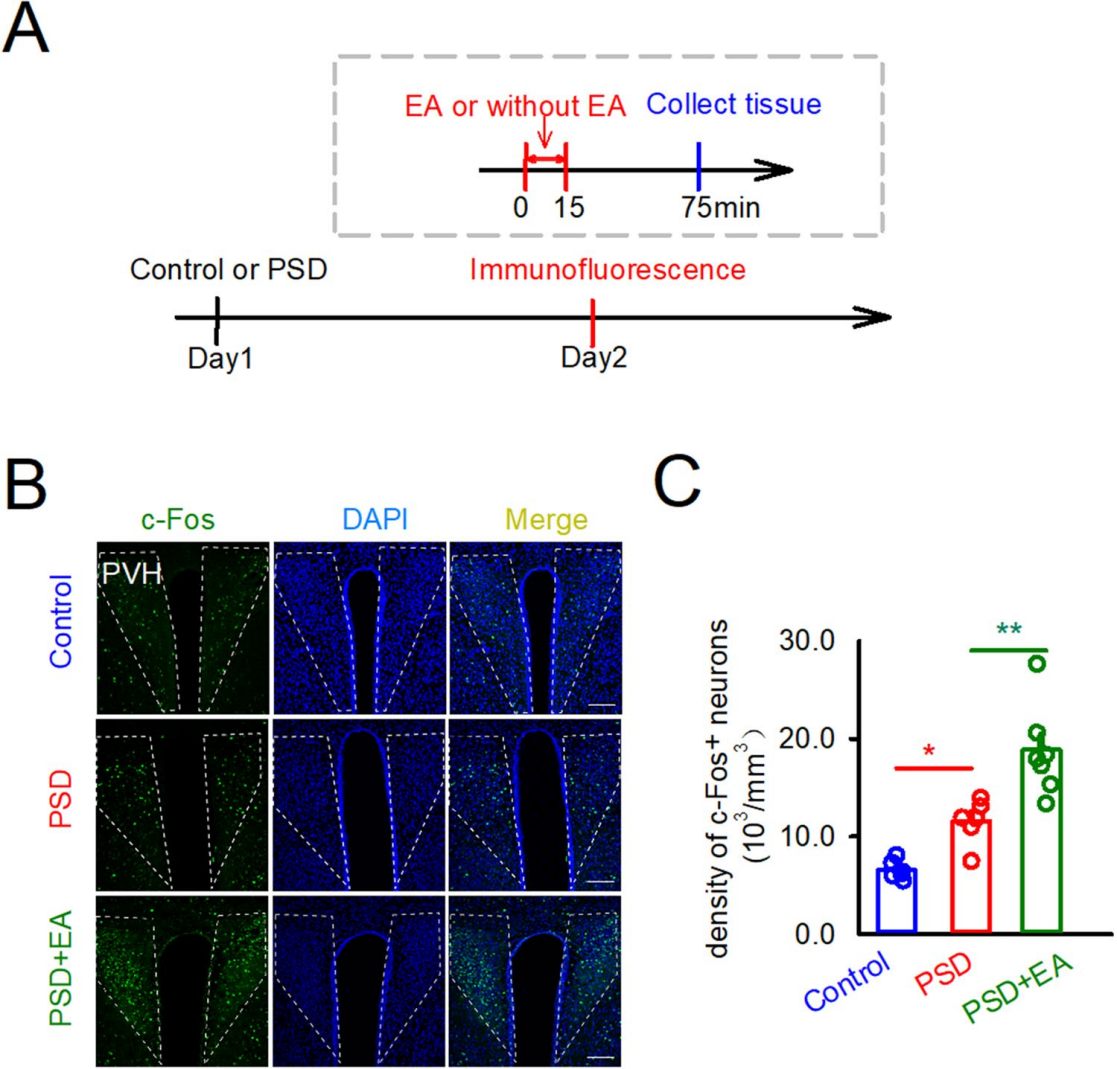
The function of PVH in stroke model. (**A**) Schematics of experimental design. (**B**) Representative images of the c-Fos neurons in PVH in the control group (top), model group (middle), model with EA at CV23 group (bottom). Data are shown as mean ± SEM (control and PSD: N = 6 slices, PSD + EA: N = 8 slices). (**C**) The statistical analysis of the density of c-Fos neurons in every group. 10^3^/mm^3^. One-way ANOVA with Tukey's multiple comparisons test, *p < 0.05, **p < 0.01. Scale bars: 100 μm.

To investigate the role of PVH in the pathological condition, the poststroke dysphagia (PSD) model was made. Focal cortical ischemia was induced by photo thrombosis of the cortical micro vessels^5, 28^. We used the photochemical method to cause infarction area in the right M1. The density of c-Fos neurons in the PVH in the model group was significantly increased compared with the control group (Control: 6580 ± 387.8, PSD: 11,540 ± 914.3, cells/mm^3^, *p* < 0.05) (Fig. 3A–C). The density of c-Fos neurons in the model group treated by EA at CV23 was clearly increased than that in the model groups (PSD: 11,540 ± 914.3, PSD + EA: 18,874 ± 1523, cells/mm^3^, *p* < 0.01) (Fig. 3A–C). To further confirm the efficacy, the sham acupoint adjacent to CV23 region was set (Fig. S2A), and the results showed EA at sham acupoint didn’t change the density of c-Fos neurons in the stroke condition (Fig. S2B&C). The c-Fos expression results indicated that neurons in the PVH could be activated by stroke induction, and further activated by EA stimulation at CV23.

### Inhibition of PVH neurons prevented the EA mediated efficacy for improving the swallowing function

**Figure 4:**
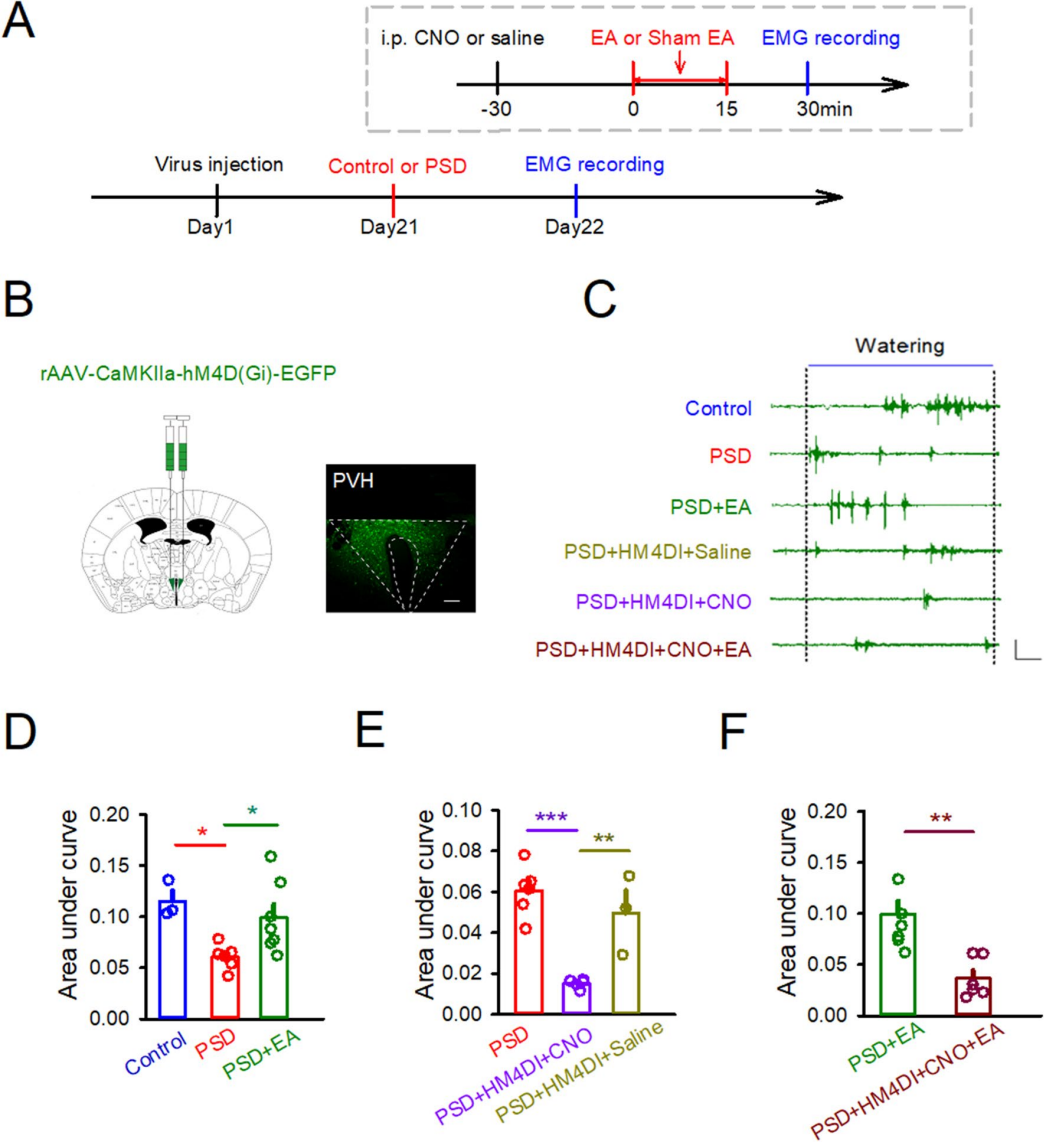
EA promoted the function of deglutition. (**A**) Schematics of experimental design. (**B**) Strategy to inhibit the PVH through inhibition of excitatory neurons. Shown is a representative image of HM4D in the PVH. Scale bars, 200 μm. (**C**) Changes in mylohyoid muscle electromyography (EMG) during a representative swallow. EMG of mylohyoid muscle in each group, Scale bars, time = 2 s, bin = 0.1mv. (D-F)The statistical analysis of the area under curve of EMG in every group. Data are shown as mean ± SEM (Control and PSD + HM4DI + Saline: N = 3 mice, PSD + HM4DI + CNO: N = 4 mice, PSD: N = 6 mice, PSD + EA: N = 7 mice, PSD + HM4DI + CNO + EA: N = 6 mice). (**D**) and (**E**) One-way ANOVA with Tukey's multiple comparisons test. (F) Unpaired t test. *p < 0.05, **p < 0.01, ***p < 0.001.

To further verify the role of excitatory neuronal cluster in the PVH in swallowing, we expressed an inhibitory (hM4D) designer receptor exclusively activated by designer drugs (DREADD) in excitatory neurons in the PVH was expressed, and this can effectively inhibit the activity of excitatory neurons (Fig. 4A,B). Pharyngeal swallows were associated with transient increases in pharyngeal pressure, contraction of the mylohyoid muscle as recorded by EMG. Treatment of mice with clozapine N-oxide (CNO) significantly attenuated swallowing response (Fig. 4C). As shown in Fig. 4D, focal cortical ischemia significantly attenuated area under the curve (AUC) of EMG responses (Control: 0.1151 ± 0.01043, PSD: 0.06066 ± 0.004932, *p* < 0.05). These results suggested that focal cortical ischemia induced dysphagia. AUC of EMG responses was also significantly elevated by EA at CV23 (PSD: 0.06066 ± 0.004932, PSD + EA: 0.09912 ± 0.01325, *p* < 0.05). However, AUC of EMG responses wasn’t changed by EA at sham acupoint (Fig. S3A&B). The result indicated that electroacupuncture at CV23 not sham acupoint improved swallowing function. The model groups with chemoinhibition the excitatory neurons in PVH injected with CNO (i.p.) significantly decreased the swallowing function compared to the model groups (PSD: 0.06066 ± 0.004932, PSD + HM4DI + CNO: 0.01521 ± 0.001004, *p* < 0.001) (Fig. 4E). However, the model groups with chemoinhibition the excitatory neurons in PVH injected with saline (i.p.) didn’t affect swallowing function (PSD + HM4DI + CNO: 0.01521 ± 0.001004, PSD + HM4DI + Saline: 0.04962 ± 0.01118, *p* < 0.01). While the inhibition of PVH didn’t elevate swallowing by EA at CV23 (PSD + EA:0.09912 ± 0.01325, PSD + EA + HM4DI + CNO: 0.03645 ± 0.007895, *p* < 0.01) (Fig. 4F). Overall, these results demonstrated the excitatory neurons in PVH involved the process of the swallowing both in the physiological and pathological condition, and also mediated the efficacy of EA at CV23.

## Discussion

The present study aimed to detect the effect of EA at CV23 on swallowing and investigated the role of excitatory neurons in PVH during this process. Our results showed that the EA at CV23 could activate excitatory neurons in PVH. The neurons in PVH both projected to and received input from many brain regions associated with food intake, including LSv, PBN and NTS^11–14^, which provides a structural basis for PVH to play a role in swallowing function. Functionally, swallowing related response was attenuated by chemoinhibition of PVH in the PSD model.

EA at CV23 was used to treat for kinds of diseases, including acute dental pain^29^, essential tremor^30^, cranial nerve palsy^31^, stroke^32^ and swallowing disorders^2, 6, 15, 33^. Acupuncture at CV23 has been evidenced to activate specific brain regions, including NA^15, 33^, VLM^2, 15^, NTS^15^, striatum^34^, central amygdala (CeA)^35^, M1^5, 36^ and S1^34, 35, 37–40^. Swallowing movements are produced by a central pattern generator (CPG) located in the lower brainstem^41^. The swallowing CPG includes two main groups of neurons located within the medulla oblongata: a dorsal swallowing group located within NTS and a ventral swallowing group located in the VLM adjacent to NA^42^. The coordination of swallowing reflex with rhythmic jaw movements could be regulated by the CeA^43^. The striatum has a prominent role in selecting which motor program should be called into action^44^. These brain regions play different roles in the swallowing process.

Our results showed that the neurons in PVH could be activated by EA stimulation at CV23, and this neuronal cluster was demonstrated as the excitatory neurons. The PVH was suggested to be composed of three main types of neurons, including magnocellular, parvocellular, and long-projecting neurons, which plays imperative roles in the regulation of energy balance and various endocrinological activities^45^. The arginine vasopressin positive neurons in the PVH participate in the regulation of feeding behaviors^46^. However, the excitatory neuron in this study belongs to which neuronal type above-mentioned is elusive. In the PVH, melanocortin-4 receptor is expressed on most glutamatergic neurons that project to PBN and dorsal motor nucleus of the vagus nerve (DMV) in the brainstem^47, 48^. Further studies would explore the specific neuronal type and clarify the role of neuronal type involved in swallowing.

The monosynaptic input and output neurons were screened throughout the whole brain. The results demonstrated that cingulate cortex, AcbSh and LSv showed strong projection to PVH (Fig. 2E). Neuronal variability in the posterior cingulate cortex (PCC) was significantly reduced after stroke^49^. The dominance of activation in contralateral cingulate cortex was associated with a better motor recovery in the early stage after stroke^27^. AcbSh sends substantial projections to at least two brain regions known to play a role in the control of feeding: the lateral hypothalamus and medial ventral pallidum^50^. LSv plays a critical role in emotionality, social behavior and feeding processes, through neural connections with the hippocampus and hypothalamus^51^. Glucagon-like peptide 1 receptors (GLP-1R) are expressed in the LSv, and endogenous GLP-1 affects feeding and motivation for food^52^. The anterior cingulate cortex (ACC) mediates the food foraging-related behaviors^53^, which is a key region underlying neural processing of social decision-making, specifically tending to compete for foraging high predictive reward food^54^. While PVH neurons both project to the PBN, they synoptically engage distinct efferent nodes, the pre-locus coeruleus and cLPBN, sufficient and necessary to control food intake^55^. The hindbrain, including NTS, PBN, and DMV, is an essential region which could integrate the feeding information from the PVH, other brain regions, and peripheral tissue^56^. Swallowing is an indispensable part during food intake. We hypothesized that these brain regions are involved in swallowing, and all these provide the basis for PVH to play a role in swallowing function.

This study provided the compelling evidence on how the excitatory neurons in PVH involved the process of EA stimulation at CV23 for regulating swallowing function. On the one hand, this result emphasized the role of excitatory neurons in PVH during the swallowing process, on the other hand, this represented new insight for understanding the mechanism of acupuncture treatment at CV23, which could guide the further clinical application of acupuncture therapy.

## Materials and methods

### Animals

C57BL/6 J mice were purchased from the Animal Laboratory Center of Guangzhou University of Chinese Medicine (license No. SCXK (Yue) 2018–0034). GAD67-GFP knock-in mice were from Professor Yongjun Chen^57^. In total, 130 mice have been used in this study as follows. Firstly, 20 mice were used in the preliminary experiments for exploring PSD molding parameters and virus injection sites. Secondly, 27 mice were used in the immunofluorescence, dividing into control group (N = 6), EA at CV23 group (N = 7), EA at ST36 group (N = 3), PSD group (N = 5) and PSD + EA group (N = 6). Thirdly, 8 mice were used in whole-brain mapping. Fourth, 45 mice were used in electromyography recording, divided into control group (N = 3), PSD group (N =9 , PSD + EA group (N = 9), PSD + HM4DI + Saline group (N = 7), PSD + HM4DI + CNO group (N = 8), and PSD + HM4DI + CNO + EA group (N = 9). We used 100 mice, presented results were obtained from 60 mice. 20 mice were excluded due to the death of mice after modeling and remaining 20 mice were used for preliminary experiments. At last, 30 mice were used to supplement the experiment for sham EA experiments, control group (N = 8), PSD group (N = 11) and PSD + Sham EA group (N = 11). Due to death of the 7 mice after modeling, presented results were obtained from 23 mice. The mice were housed in cages (5 mice/cage) with an ambient temperature of 25 ± 2 °C, a 12-h light–dark cycle, and ad libitum access to water and food. Animal care and experimental manipulation were conducted in accordance with the National Institutes of Health Guide for the Care and Use of Laboratory Animals and approved by the Committee for Care and Use of Research Animals of Guangzhou University of Chinese Medicine (approval No. 20170303), March 3, 2017. The study was carried out in compliance with the ARRIVE guidelines.

### PSD modeling

Mice were anesthetized with intraperitoneally (i.p.) injected Tribromoethanol (1.25%, 125 mg/kg Sigma–Aldrich, Saint Louis, MO, USA). Ten minutes prior to illumination, Rose Bengal solution (1.5%, Sigma–Aldrich, Saint Louis, MO, USA, 100 mg/kg, i.p.) was administered. Mice were fixed on stereotaxic apparatus (RWD Biotechnology, Shenzhen, China). The skin was cut, following the skull was exposed, and a fiber optic cable delivering laser light was placed onto the skull using a stereotaxic frame. The laser light was centered 0.13 mm anterior to the bregma and 1.1 mm lateral from the midline, targeting the right motor cortex (AP: -0.13, ML: 1.1). A laser (wave length: 530 nm; power: 6.5 mw) was used to irradiate an area of ~ 2mm^2^. After 7 min of irradiation, the scalp was sutured, and mice were placed back in the home cage to recover from anesthesia. Swallowing-related muscle functions were evaluated in awake mice.

### EA treatment

EA was applied at CV23, ST36 and sham acupoint (located at the right side of cervical region) for 15 min, respectively, and the control group did not take any treatment. Anesthesia was induced using 4% isoflurane, and maintained with 2% isoflurane using a mask. After routinely sterilized neck skin, we inserted an acupuncture needle to the upper margin of the midline of the mandible, where CV23 acupoint located. Another needle was inserted 2 mm adjacent to CV23. The needling depth of CV23 is 5 mm. In mouse, ST36, located at 3 mm below the fibular head, in the posterolateral part of the knew. The EA apparatus (continuous wave; current, 1 mA; frequency, 2 Hz; time,15 min per day; HANS-200A/100B; HANS, Beijing, China) was attached to the acupuncture needle. EA acupuncture was treated for once.

### Perfusion and tissue sectioning

c-Fos immunoreactivity was investigated in GAD67-GFP and C57 mice following EA treatment. One hour after the treatment, mice were anesthetized with injected (i.p.) Tribromoethanol (125 mg/kg) and perfused transcardially with 0.9% cold saline (Macklin; China) followed by 4% paraformaldehyde (PFA) (mubiotech, China) in PBS (Thermo Fisher Scientific, America). The brains were extracted, postfixed overnight in 4% PFA at 4 °C and cryopreserved in 15% and 30% sucrose in PBS, and then embedded in Tissue-Tek OCT (Sakura, Japan) compound. Each brain (40 μm) was cut in the transverse plane using a freezing microtome (Thermo, Micro International GmbH, Germany).

### c-Fos immunostaining

Free-floating sections were washed in PBS, incubated for 1 h in blocking solution (1% BSA (Macklin; China) at 37 °C and 0.3% Triton X-100 (Biosharp, China) in PBS and incubated overnight at 4 °C with primary antibodies (see below for a list of antibodies) in blocking solution. For c-Fos staining, slices were incubated with the primary antibody for one night at 4 °C. Sections were then washed with PBS and incubated for 2 h at 37 °C with secondary antibodies (see below for a full list of antibodies) in 0.3% Triton X-100 in PBS. Finally, sections were washed in PBS, incubated with 4,6-diamidino-2-phenylindole (DAPI:1 µg/5 ml, Sigma, America) and mounted on slides with mounting medium (50% Glycerol anhydrous in PBS, Biofroxx, Germany).

### Antibodies

Primary antibodies. Mouse anti-NeuN (millipore, catalog no. 3075598; diluted 1:500); and rabbit anti c-Fos (Cell Signaling Technology, catalog no. 2250; diluted 1:500).

Secondary antibodies. All from donkey anti-rabbit (conjugated to Alexa Fluor 488, catalog no. 711–545-152; diluted 1:500); donkey anti- rabbit (conjugated to life 594, catalog no. A21207; diluted 1:500), goat anti-mouse (Alexa Fluor 594, catalog no. ab150116; diluted 1:500).

### Confocal microscopy

Confocal fluorescence images (Nikon, Japan) were acquired on a Nikon scanning laser microscope using × 20 and × 40 air objectives. Image analysis was performed using either ImageJ (1.52a, National Institutes of Health, America) or NIS-Viewer v.4.5 (Nikon, Japan).

### Quantification and analysis

Expression of c-Fos was manually quantified at various brain regions by an observer blind to the mouse’s experimental condition, using a brain atlas for guidance. Area was measured using ImageJ software (1.52a, National Institutes of Health, America). Next, the PVH (median eminence not included) was contoured and the area was measured on all images based on the labelling for c-Fos. The reference volume was determined as the sum of the area multiplied by the distance between sampled sections (40 μm). The c-Fos positive cell density was calculated for each slice by dividing the total sampled cell numbers by the total volume of the region. A schematic diagram of the selected brain regions is shown in Figs. 1, 3, S2.

### Electromyography recording in the Mylohyoid Muscle In Vivo

Anesthesia was induced with 4% isoflurane (Sigma, America). Mice were fixed supine on stereotaxic apparatus. A tube (0.85*0.42 mm PE tube) was adjusted for placement under the tongue. A recording electrode was inserted into the mylohyoid muscle. The reference electrode was inserted into the masseter muscle. The EMG activity of the mylohyoid muscle was evoked and acquired with the Spike2 software (CED, Cambridge, UK) when mice were given water (2 μl/s, 10 s) by a micro-injection pump (HARVARD, USA) after waking up from anesthesia. Data was digitized at 5 kHz with a Power 1401 digitizer (CED, UK) and band-pass filtered at 0.1–1 kHz with a 1902 differential AC amplifier (CED, UK). Evaluated parameters of EMG response is the area under the curve (AUC, represented by mV* msec) of integrated^58^. The area under the curve was calculated in a 10 s window from the onset of water delivery in EMG analysis.

### Stereotaxic surgery

Mice were anesthetized with injected (i.p.) Tribromoethanol (125 mg/kg) and fixed on stereotaxic apparatus. After incision, the skull was exposed and holes were drilled over the targeted areas at the following coordinates from bregma. Virus or CTB was injected at a rate of 30 nl/min. After 10 min, the needle was retracted and the incision was closed with a sterile suture. For chemogenomic experiments, rAAV-CaMKIIα-hM4D(Gi)-EGFP (300 nl) was bilateral injected in PVH for inhibition. rAAV-CaMKII-mCherry (50 nl) was unilateral injected in PVH for tracing. Mice were given about 3 weeks to allow expression before next manipulation. CTB-488 (50 nl) was unilateral injected in PVH for afferent trace. CTB was injected 5 days prior to sacrifice for retrograde transport. The viruses or CTB-488 were injected according to the following coordinates: PVH (AP: – 0.82; ML: ± 0.2; DV: – 4.9). Coordinate mapping and injection was performed using stereotaxic apparatus (RWD Biotechnology, Shenzhen, China).

### CNO-induced inhibition

DREADD-related experiments were performed after waiting 3 weeks for viral expression. Mice were briefly anesthetized with isoflurane then fixed supine on stereotaxic apparatus. We recorded the EMG activity of the mylohyoid muscle at 30 min after clozapine N-oxide (CNO) injection (1 mg/kg, i.p.).

### Statistical analyses

All data analysis was performed using Sigmaplot (version14.0) and Prism 7.0 (GraphPad Software). Data were shown as means ± SEM. The differences between groups were analyzed by a one-way analysis of variance (ANOVA) or Unpaired t test, statistical significance was defined as *p* < 0.05 *, *p* < 0.01 ** and *p* < 0.001 ***.

### Ethics approval and consent to participate

Approval was obtained from the ethics committee of Guangzhou University of Chinese Medicine.

### Consent for publication

The authors have provided consent for publication of the article.

## Supplementary Information

Supplementary Figures.

## Data Availability

The data and material discussed here are available in the references listed.

## Abbreviations

CeA: Central amygdala
CGPn: Hypothalamus, central gray of the pons
CNO: Clozapine N-oxide
cLPBN: Central lateral parabrachial nucleus
CPG: Central pattern generator
CPu: Caudate putamen (striatun)
CV23: Lianquan
Cg2: Cingulate cortex, area2
DMV: Dorsal motor nucleus of the vagus nerve
EA: Electroacupuncture
EMG: Electromyography
Lsv: Ventral part of the lateral septal nucleus
M1: The primary motor cortex
NA: Nucleus ambiguous
NTS: The solitary tract nucleus
PBN: Parabrachial nucleus
PSD: Poststroke dysphagia
PVH: Paraventricular hypothalamus
S1: The primary sensory cortex
ST36: Zusanli
VDB: Nucleus of the vertical limb of the diagonal band
VLM: Ventrolateral medulla

## References

[1] Bath PM, Lee HS, Everton LF (2018). Swallowing therapy for dysphagia in acute and subacute stroke. Cochrane Database Syst. Rev..

[2] Ye Q (2019). Effect of electro-acupuncture on regulating the swallowing by activating the interneuron in ventrolateral medulla (VLM). Brain Res. Bull..

[3] Han CH (2020). Electroacupuncture for post-stroke dysphagia: A protocol for systematic review and meta-analysis of randomized controlled trials. Medicine.

[4] Huang J (2020). Clinical effects and safety of electroacupuncture for the treatment of poststroke dysphagia: A comprehensive systematic review and meta-analysis. Evidence-Based Complement. Altern. Med. eCAM.

[5] Yao S (2020). Effect of different frequencies of electroacupuncture on post-stroke dysphagia in mice. J. Mol. Neurosci. MN.

[6] Qin L (2019). Deep acupuncture of Lianquan (CV23) and Yifeng (TE17) in combination with conventional acupuncture of other acupoints is superior to swallowing rehabilitation training in improving post-stroke dysphagia in apoplexy patients. Zhen ci yan jiu = Acupunct. Res..

[7] Fontana JM (2015). Energy intake estimation from counts of chews and swallows. Appetite.

[8] Fukatsu H (2015). Endoscopic evaluation of food bolus formation and its relationship with the number of chewing cycles. J. Oral Rehabil..

[9] Sweeney P, Li C, Yang Y (2017). Appetite suppressive role of medial septal glutamatergic neurons. Proc. Natl. Acad. Sci. U S A.

[10] Mangieri LR (2019). Defensive behaviors driven by a hypothalamic-ventral midbrain circuit. eNeuro.

[11] Varela L, Horvath TL (2019). Parallel paths in PVH control of feeding. Neuron.

[12] Cabral A (2020). Fasting induces remodeling of the orexigenic projections from the arcuate nucleus to the hypothalamic paraventricular nucleus, in a growth hormone secretagogue receptor-dependent manner. Mol. Metabol..

[13] Xu Y (2019). Identification of a neurocircuit underlying regulation of feeding by stress-related emotional responses. Nat. Commun..

[14] Roman CW, Sloat SR, Palmiter RD (2017). A tale of two circuits: CCK(NTS) neuron stimulation controls appetite and induces opposing motivational states by projections to distinct brain regions. Neuroscience.

[15] Cui S (2020). Electroacupuncture involved in motor cortex and hypoglossal neural control to improve voluntary swallowing of poststroke dysphagia mice. Neural Plast.

[16] Perrin-Terrin AS (2016). The c-FOS protein immunohistological detection: A useful tool as a marker of central pathways involved in specific physiological responses in vivo and ex vivo. J. Vis. Exp..

[17] Bonis JM (2013). Contributions of the Kölliker-Fuse nucleus to coordination of breathing and swallowing. Respir. Physiol. Neurobiol..

[18] Tsujimura T, Inoue M (2020). Evaluation of the association between orofacial pain and dysphagia. J. Oral. Sci..

[19] Lang IM (2009). Brain stem control of the phases of swallowing. Dysphagia.

[20] Liu AKL (2018). Review: Revisiting the human cholinergic nucleus of the diagonal band of Broca. Neuropathol. Appl. Neurobiol..

[21] de la Roza C, Reinoso-Suárez F (2006). GABAergic structures in the ventral part of the oral pontine reticular nucleus: An ultrastructural immunogold analysis. Neuroscience.

[22] Erthal V, Maria-Ferreira D, Werner MF, Baggio CH, Nohama P (2016). Anti-inflammatory effect of laser acupuncture in ST36 (Zusanli) acupoint in mouse paw edema. Lasers Med. Sci..

[23] Zhang H (2018). Laser stimulating ST36 with optical fiber induce blood component changes in mice: A Raman spectroscopy study. J. Biophoton..

[24] Ferguson AV, Latchford KJ, Samson WK (2008). The paraventricular nucleus of the hypothalamus-a potential target for integrative treatment of autonomic dysfunction. Expert Opin. Ther. Targets.

[25] Hossain MZ, Ando H, Unno S, Kitagawa J (2020). Targeting chemosensory ion channels in peripheral swallowing-related regions for the management of oropharyngeal dysphagia. Int. J. Mol. Sci..

[26] Browaldh N, Bautista TG, Dutschmann M, Berkowitz RG (2016). The Kolliker-Fuse nucleus: A review of animal studies and the implications for cranial nerve function in humans. Eur. Arch. Otorhinolaryngol..

[27] Li Y (2016). Functional lateralization in cingulate cortex predicts motor recovery after basal ganglia stroke. Neurosci. Lett..

[28] Jander S, Schroeter M, Stoll G (2002). Interleukin-18 expression after focal ischemia of the rat brain: Association with the late-stage inflammatory response. J. Cerebral Blood Flow Metabol. Off. J. Int. Soc. Cerebral Blood Flow Metabol..

[29] Grillo CM, Wada RS, da Luz R, de Sousa M (2014). Acupuncture in the management of acute dental pain. J. Acupuncture Meridian Stud..

[30] Jeong JJ, Sun SH (2013). Sa-am five-element acupuncture and hwangyeon- haedoktang pharmacopuncture treatment for an essential tremor: Three case reports. J. Pharmacopunct..

[31] Sun SH (2012). Idiopathic ninth, tenth, and twelfth cranial nerve palsy with ipsilateral headache: A case report. J. Pharmacopunct..

[32] Yuan Y (2019). Clinical trials of acupuncture treatment of post-stroke dysphagia by deep acupuncture of Tiantu (CV22) in combination with swallowing rehabilitation training. Zhen ci yan jiu = Acupuncture Res..

[33] Shi J (2019). EA promotes swallowing via activating swallowing-related motor neurons in the nucleus ambiguus. Brain Res..

[34] Li Z (2020). Electroacupuncture promotes motor function and functional connectivity in rats with ischemic stroke: An animal resting-state functional magnetic resonance imaging study. Acupuncture Med. J. Br. Med. Acupuncture Soc..

[35] Xu Z (2020). Electroacupuncture alleviates pain-related emotion by upregulating the expression of NPS and its receptor NPSR in the anterior cingulate cortex and hypothalamus. Evidence-Based Complement. Alternative Med. eCAM.

[36] Sun ZG (2019). Effect of acupuncture at ST36 on motor cortical excitation and inhibition. Brain Behav..

[37] Cha M, Chae Y, Bai SJ, Lee BH (2017). Spatiotemporal changes of optical signals in the somatosensory cortex of neuropathic rats after electroacupuncture stimulation. BMC Complement. Altern. Med..

[38] Lee DY, Jiu YR, Hsieh CL (2020). Electroacupuncture at Zusanli and at Neiguan characterized point specificity in the brain by metabolomic analysis. Sci. Rep..

[39] Liao ET, Tang NY, Lin YW, Liang Hsieh C (2017). Long-term electrical stimulation at ear and electro-acupuncture at ST36-ST37 attenuated COX-2 in the CA1 of hippocampus in kainic acid-induced epileptic seizure rats. Sci. Rep..

[40] Ye Y (2017). Acupuncture attenuated vascular dementia-induced hippocampal long-term potentiation impairments via activation of D1/D5 receptors. Stroke.

[41] Inoue M (2015). The neural mechanisms underlying swallowing. Brain Nerve = Shinkei kenkyu no shinpo.

[42] Jean A (2001). Brain stem control of swallowing: Neuronal network and cellular mechanisms. Physiol. Rev..

[43] Satoh Y, Tsuji K (2020). Suppression of the swallowing reflex during rhythmic jaw movements induced by repetitive electrical stimulation of the dorsomedial part of the central amygdaloid nucleus in rats. Life (Basel, Switzerland).

[44] Grillner S, Hellgren J, Ménard A, Saitoh K, Wikström MA (2005). Mechanisms for selection of basic motor programs–roles for the striatum and pallidum. Trends Neurosci..

[45] Qin C, Li J, Tang K (2018). The paraventricular nucleus of the hypothalamus: Development, function, and human diseases. Endocrinology.

[46] Pei H, Sutton AK, Burnett KH, Fuller PM, Olson DP (2014). AVP neurons in the paraventricular nucleus of the hypothalamus regulate feeding. Mol. Metabol..

[47] Xu Y (2013). Glutamate mediates the function of melanocortin receptor 4 on Sim1 neurons in body weight regulation. Cell Metab..

[48] Garfield AS (2015). A neural basis for melanocortin-4 receptor-regulated appetite. Nat. Neurosci..

[49] Farinelli M (2020). Brain and behaviour in post-acute stroke: Reduction in seeking and posterior cingulate neuronal variability. J. Clin. Exp. Neuropsychol..

[50] Stratford TR, Wirtshafter D (2012). Evidence that the nucleus accumbens shell, ventral pallidum, and lateral hypothalamus are components of a lateralized feeding circuit. Behav. Brain Res..

[51] Deng K (2019). Whole-brain mapping of projection from mouse lateral septal nucleus. Biol. Open.

[52] Terrill SJ (2019). Endogenous GLP-1 in lateral septum promotes satiety and suppresses motivation for food in mice. Physiol. Behav..

[53] Li F (2012). Anterior cingulate cortical lesion attenuates food foraging in rats. Brain Res. Bull..

[54] Zhong X (2017). Anterior cingulate cortex involved in social food-foraging decision-making strategies of rats. Brain Behav..

[55] Li MM (2019). The paraventricular hypothalamus regulates satiety and prevents obesity via two genetically distinct circuits. Neuron.

[56] Grill HJ, Hayes MR (2012). Hindbrain neurons as an essential hub in the neuroanatomically distributed control of energy balance. Cell Metab..

[57] Ting AK (2011). Neuregulin 1 promotes excitatory synapse development and function in GABAergic interneurons. J. Neurosci..

[58] Su X, Cutinella M, Koppes S, Agran JE, Dinsmoor DA (2019). Electromyographic responses across different pulse-widths of sacral neuromodulation in sheep. Neuromodulation.

