# Supplementary material for: Excitatory neurons in paraventricular hypothalamus contributed to the mechanism underlying acupuncture regulating the swallowing function
Source: Sci Rep. 2022 Apr 6;12:5797. doi: 10.1038/s41598-022-09470-9 (PMC8987055; doi:10.1038/s41598-022-09470-9)
Supplement: Supplementary file 1 — Supplementary Figures. [file 41598_2022_9470_MOESM1_ESM.docx]

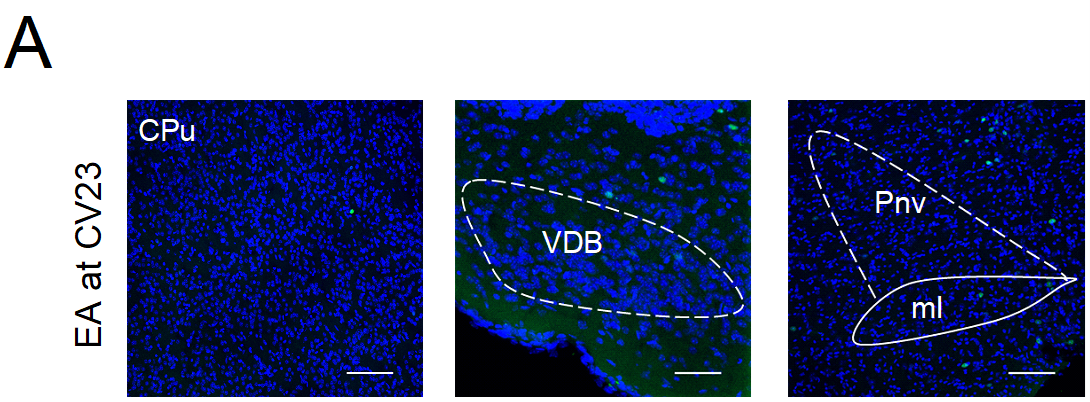
**Supplementary figure1:**

**Supplementary figure1:**

(A) Confocal images showing c-Fos expression in CPu (left), VDB (middle), Pnv and ml (right). EA at CV23 group. Middle: Scale bars, 50μm, left and right: scale bars, 100μm.


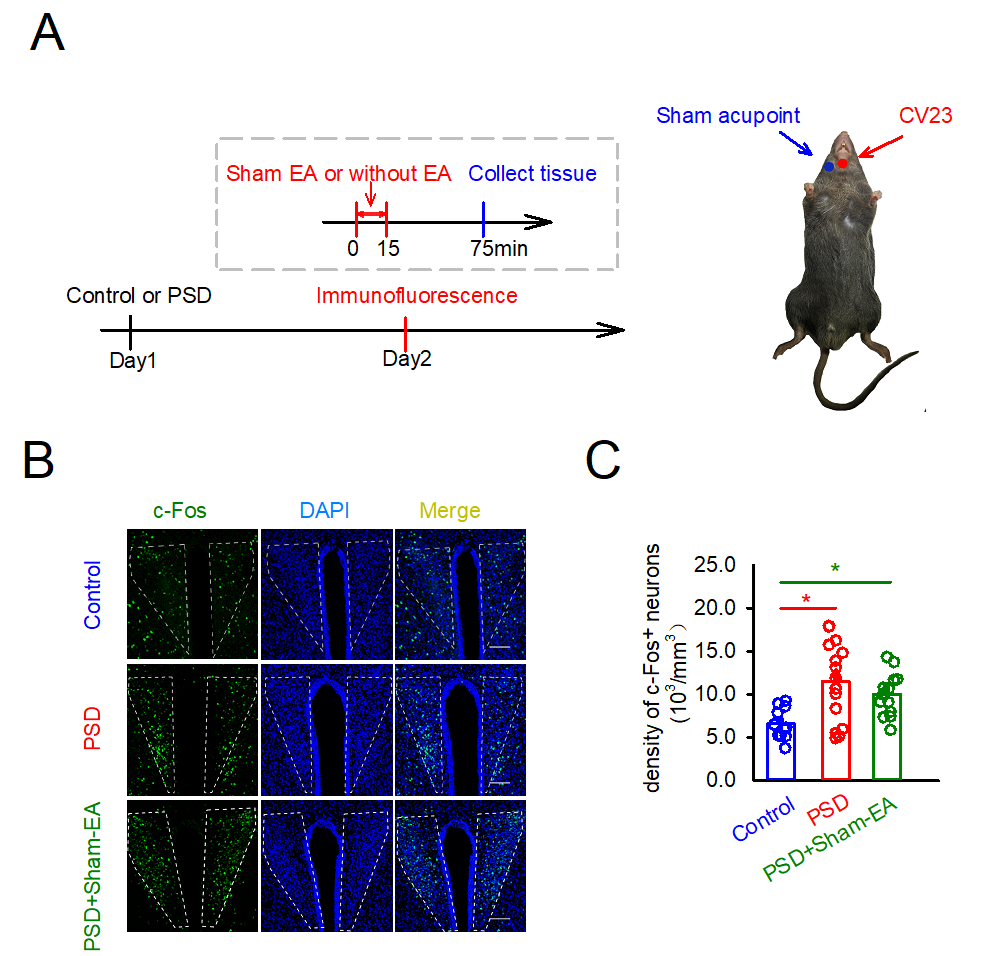
**Supplementary figure2：**

Supplementary figure2：

(A) Schematics of experimental design and schematics showing the sham acupoint. (B) Representative images of the c-Fos neurons in PVH in the control group (top), model group (middle), model with EA at sham acupoint group (bottom). Data are mean ± SEM (control: N= 11 slices, PSD and PSD+EA: N= 15 slices, respectively). (C) The statistical analysis of the number density of c-Fos neurons in every group. 10^3^/mm^3^. Scale bars: 100μm. One-way ANOVA with Tukey's multiple comparison test, **p* < 0.05.


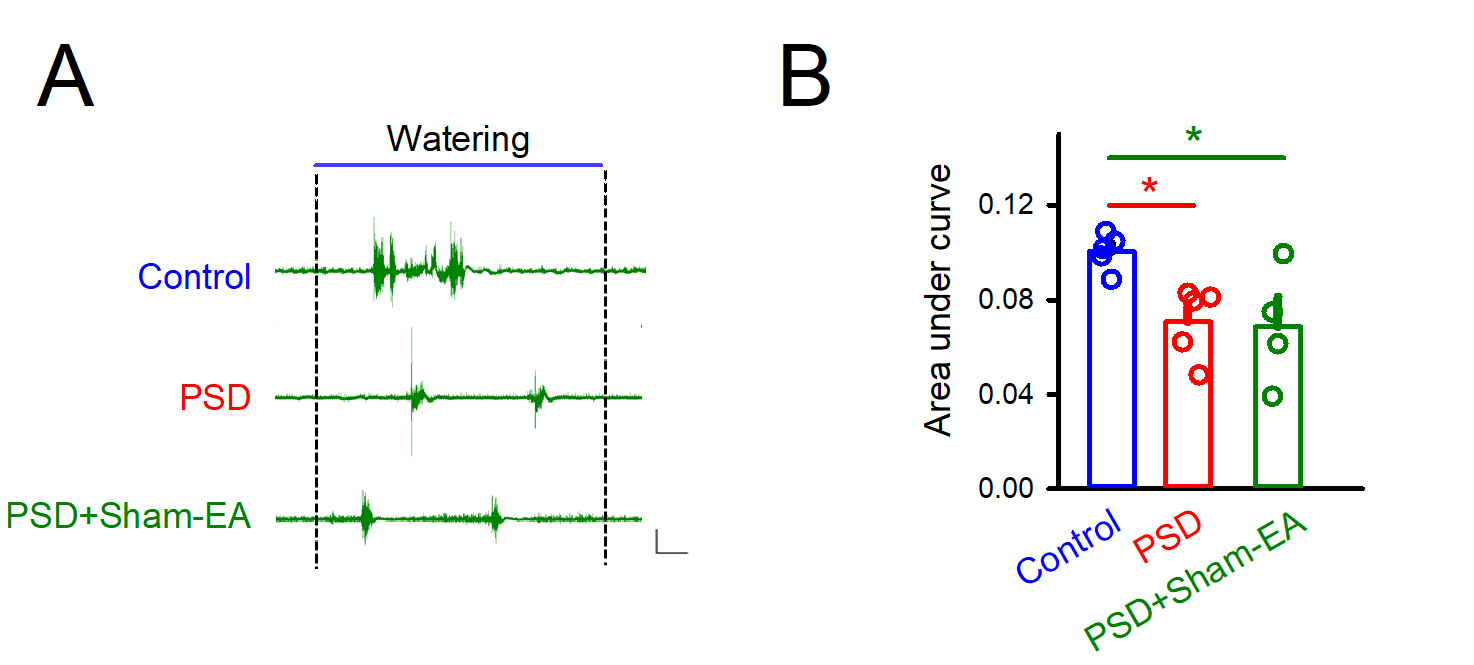
**Supplementary figure3：**

**Supplementary figure3:**

(A) Changes in mylohyoid muscle electromyography (EMG) during a representative swallow. EMG of mylohyoid muscle in every group, time = 2s, bin = 0.1mv. (B) The statistical analysis of the area under curve of EMG in every group. Data are mean ± SEM（Control: N= 5 mices, PSD: N= 5 mices, PSD + Sham-EA: N= 4 mices). One-way ANOVA with Tukey's multiple comparison test, **p*< 0.05.
